# Application of deep learning with bivariate models for genomic prediction of sow lifetime productivity-related traits

**DOI:** 10.5713/ab.23.0264

**Published:** 2024-01-14

**Authors:** Joon-Ki Hong, Yong-Min Kim, Eun-Seok Cho, Jae-Bong Lee, Young-Sin Kim, Hee-Bok Park

**Affiliations:** 1 Swine Division, National Institute of Animal Science, Rural Development Administration, Cheonan 31000, Korea; 2 Korea Zoonosis Research Institute, Jeonbuk National University, Iksan 54531, Korea; 3 Department of Animal Resources Science, Kongju National University, Yesan 32439, Korea; 4 Resource Science Research Institute, Kongju National University, Yesan 32439, Korea

**Keywords:** Convolutional Neural Network, Deep Learning, Epistatic Interaction, Genomic Prediction, Pig, Sow Lifetime Productivity

## Abstract

**Objective:**

Pig breeders cannot obtain phenotypic information at the time of selection for sow lifetime productivity (SLP). They would benefit from obtaining genetic information of candidate sows. Genomic data interpreted using deep learning (DL) techniques could contribute to the genetic improvement of SLP to maximize farm profitability because DL models capture nonlinear genetic effects such as dominance and epistasis more efficiently than conventional genomic prediction methods based on linear models. This study aimed to investigate the usefulness of DL for the genomic prediction of two SLP-related traits; lifetime number of litters (LNL) and lifetime pig production (LPP).

**Methods:**

Two bivariate DL models, convolutional neural network (CNN) and local convolutional neural network (LCNN), were compared with conventional bivariate linear models (i.e., genomic best linear unbiased prediction, Bayesian ridge regression, Bayes A, and Bayes B). Phenotype and pedigree data were collected from 40,011 sows that had husbandry records. Among these, 3,652 pigs were genotyped using the PorcineSNP60K BeadChip.

**Results:**

The best predictive correlation for LNL was obtained with CNN (0.28), followed by LCNN (0.26) and conventional linear models (approximately 0.21). For LPP, the best predictive correlation was also obtained with CNN (0.29), followed by LCNN (0.27) and conventional linear models (approximately 0.25). A similar trend was observed with the mean squared error of prediction for the SLP traits.

**Conclusion:**

This study provides an example of a CNN that can outperform against the linear model-based genomic prediction approaches when the nonlinear interaction components are important because LNL and LPP exhibited strong epistatic interaction components. Additionally, our results suggest that applying bivariate DL models could also contribute to the prediction accuracy by utilizing the genetic correlation between LNL and LPP.

## INTRODUCTION

Sow lifetime productivity (SLP)-related traits are essential for production efficiency and profitability [1–3]. To ensure profitability, a sow should produce at least three litters before being culled [4]. Early culling results in fewer piglets born alive over the sow’s lifetime and leads to irregular replacement of sows. Therefore, lifetime production traits, such as lifetime number of litters (LNL) or lifetime pigs production (LPP), are important because of their association with stayability, productivity, and animal welfare [5–7]. Additionally, predicting the breeding values of these traits at an early age is required because the breeder can only know the phenotypes of these sows after culling.

Estimating genomic breeding values using genome-wide single nucleotide polymorphism (SNP) data is vital for selection in livestock breeding [8]. Over the years, various linear model-based methods for predicting genomic breeding values have been proposed. Methods applied successfully include genomic best linear unbiased prediction (GBLUP) and Bayesian models, such as Bayesian ridge regression (BRR), Bayes A, and Bayes B [9,10]. Additionally, multi-trait models applied with these approaches can increase the accuracy of genomic breeding values compared with single-trait genomic prediction models by considering genetic correlations among the traits of interests [11–13].

With recent advances in the computational power, more researchers are applying deep learning (DL) methods such as convolutional neural networks (CNN) in genetic and behavioral studies [14–17].

However, whether DL is superior to conventional linear model-based genomic prediction models is unclear [18]. Nevertheless, in genomic prediction, DL can be conducted without specific genetic model assumptions. A DL technique can robustly analyze a phenotype of interests governed by additivity, dominance or epistasis because it admits linear and numerous nonlinear activation functions [18,19].

Several studies have been conducted using DL characteristics in genomic prediction [20–22]. Furthermore, to enhance prediction accuracy in genomic selection, improved DL models have been introduced using the multi-trait frameworks that utilize genetic correlations between the traits [23–25].

Many DL studies in genomic prediction have been mostly conducted in plant breeding [26–30]. However, only a limited number of DL studies have been conducted in livestock. In particular, DL studies in pigs have mainly focused on the number of live-born piglets [21,31]. Hence, further investigations and verifications of DL models with real data are required in pigs. Several DL models have been developed; however, among them, the CNN model is one of the most utilized DL algorithms in various fields [32]. The CNN has an additional convolutional layer and a flattend layer in front of the fully-connected layers without pooling. In each convolutional layer, the CNN automatically performs the convolution operation along with an input of a predefined width and strides through the application of kernels for all SNP marker windows [19]. Local convolutional neural network (LCNN) includes a local convolutional layer instead of the convolutional layer of a CNN. The LCNN provides a natural extension to convolutional layers by applying a region-specific filter [33]. The CNN assigns weight parameters applied to the same filter across all SNPs, whereas the LCNN fits them independently from each other. Thus, LCNN can correspond a lot better with our prior genetic knowledge of the genetic architecture of traits than traditional CNN [33].

In this study, we aimed to investigate the usefulness of the DL technique for the bivariate genomic prediction of SLP-related traits in pigs. The target traits of this study were SLP-related LNL and LPP. We optimized the hyper-parameters of two DL models (i.e., CNN and LCNN) and compared them to the conventional linear models (i.e., GBLUP, BRR, Bayes A, and Bayes B). The predictive ability of the different methods was assessed as the correlation and the mean squared error (MSE) between the predicted and observed phenotypes using five-fold cross-validation.

## MATERIALS AND METHODS

All experimental protocols were approved (approval number: NIAS20191511) by the Institutional Animal Care and Use Committee (IACUC) of the National Institute of Animal Science, Republic of Korea. All methods in this study were performed according to the relevant guidelines and regulations.

### Dataset

We used datasets of the Large White and Landrace pigs that were born and raised at breeding farms in the Republic of Korea. These farms are well connected to a few farms for each breed as a national swine genetic evaluation system in Korea [34,35].

Most pigs were born during the 2005 through 2017 decade. The phenotypic database included records of 40,011 sows with LNL and LPP. LNL ranged from 1 to 8, with a mean value of 4.5, and LPP ranged from 1 to 133, with a mean value of 48. For further analyses, adjusted means were calculated using the residuals derived separately for the individual environmental factors [18,21,36]. For adjustment, the effects of breed (Large White or Landrace), sow birth year (2005 through 2017), and herd-year-season at last farrowing (270 levels) were fitted as fixed effects. Finally, a total of 3,652 adjusted phenotypes derived from Korean Large White (n = 2,629) and Landrace (n = 1,023) sows with high-density genotypes were used for the purpose of genomic evaluation.

Genotyping was performed using the Illumina PorcineSNP60 BeadChip (Illumina, San Diego, CA, USA). The quality control process for the genotype data included the removal of individuals with pedigree errors, omission of monomorphic SNP genotypes, SNPs on sex chromosomes, SNPs with minor allele frequencies (<0.95), genotype call rate of <0.90, animal missing rate of >0.90. After quality control 47,335 SNPs remained. Missing genotypes were imputed with the FImpute program using pedigree information [37].

### Estimation of variance components and genetic parameters

Since genetic architecture can influence the optimal model choice for genomic prediction and the accuracy, additive and non-additive genetic effects on the two SLP-related traits were evaluated by estimating variance components using the following bivariate linear mixed model:


$$\left[\begin{array}{c} y_{1}\\ y_{2} \end{array}\right]=\left[\begin{array}{cc} I_{1} & 0\\ 0 & I_{2} \end{array}\right]\;\left[\begin{array}{c} \mu_{1}\\ \mu_{2} \end{array}\right]+\left[\begin{array}{cc} Z_{1} & 0\\ 0 & Z_{2} \end{array}\right]\;\left[\begin{array}{c} a_{1}\\ a_{2} \end{array}\right]+\left[\begin{array}{cc} Z_{1} & 0\\ 0 & Z_{2} \end{array}\right]\;\left[\begin{array}{c} d_{1}\\ d_{2} \end{array}\right]+\left[\begin{array}{cc} Z_{1} & 0\\ 0 & Z_{2} \end{array}\right]\;\left[\begin{array}{c} aa_{1}\\ aa_{2} \end{array}\right]+\left[\begin{array}{c} e_{1}\\ e_{2} \end{array}\right]$$

where **y_1_** and **y_2_** are the vectors of the measured phenotypes for the LNL and LPP phenotypes, ***μ*_1_** and ***μ*_2_** are the vectors of population means for the two traits, **a_1_** and **a_2_** are the vectors of random additive genetic effects for the two traits, **d_1_** and **d_2_** are the vectors of random dominance effects for the two traits, **aa_1_** and **aa_2_** are the vectors of random epistatic effects (second-order nonlinear interaction in this study) for the two traits, **e_1_** and **e_2_** is the vectors of random residuals. **Z_1_** and **Z_2_** are the are the incidence matrices relating observations with random additive, dominance and epistatic effects. The random genetic effects a, d, δ were assumed as multivariate normal distribution (MVN) given respectively by **a**~MVN(0,**G_a_**) where **G_a_** = **A⊗V_a_**, **d**~MVN(0,**G_d_**) where **G_d_** = **D⊗V_d_**, **aa**~MVN(0,**G_aa_**) where **G_aa_** = **AA⊗V_aa_** and **e**~MVN(0,**R**) where **R** = **R_0_⊗I**. The matrices were obtained by the Kronecker product (**⊗**). The additive genetic relationship matrix (**A**) was constructed using the genome-wide SNP marker data [38] as follows:


$$A=\frac{{\mathrm{ZZ}}^{\prime }}{\Sigma \;2\;p_{i}q_{i}}$$

where **Z** is a matrix that accommodates the centered individual SNP marker genotype values, *p_i_* is the frequency of the reference allele, and *q_i_* is the frequency of the alternative allele at the *i*th SNP marker.

To construct a dominance relationship matrix (**D**) using SNP marker information, we created an incidence matrix **H**, which contains the matrix of heterozygosity coefficients with element h_ki_ = 1−2*p_i_q_i_* when individual k is heterozygous at the ith SNP marker, and hki = 0−2p_i q_i in other cases [39]. Consequently,


$$d=\frac{{\mathrm{HH}}^{\prime }}{\Sigma \;2\;p_{i}q_{i}(1-2p_{i}q_{i})}$$

The epistatic genetic relationship matrix (**AA**) can be derived from the Hadamard products and traces of additive genetic relationship matrices [40],


$$\mathrm{AA}=\frac{A⊙A}{\mathrm{tr}(A⊙A)/n}$$

**V_a_**, **V_d_**, **V_a_**, and **R_0_** are unstructured 2×2 matrices. **I** is an identity matrix. To estimate the genetic correlation coefficient, we considered only the additive genetic (co)variance components. Genetic parameters were estimated in a Bayesian framework using the BGLR R-package [41]. The model was run using a Gibbs sampler algorithm for a total of 110,000 cycles, discarding the first 10,000 samples for burn-in.

### Design of linear models for genomic prediction

All linear models were implemented using the multi-trait method in the BGLR R-package that applies Bayesian inference [41]. We evaluated the performance of four different models (i.e., GBLUP, BRR, Bayes A, and Bayes B). The GBLUP and BRR models were implemented using the multi-trait function. For the Bayes A and B models, we used the BGLR function for each trait and predicted genomic breeding values with a multi-trait method using singular value decompositions [42]. The basic bivariate linear model is as follows:


y=μ1+Zg+e

where **y** is the vector of adjusted LNL and LPP phenotypes, ***μ*1** is the vector of means for the two phenotypes, **g** is the vector with the effects of the markers, assumed to be random, whose assumptions depend on the model used (i.e., additive vs. additive + dominance + epistasis) **e** is the vector of residuals, and **Z** is the incidence matrix of the genotypes.

GBLUP assigns the same variance to all loci and treats them all as equally important in a genomic relationship matrix [38]. BRR is a Bayesian method in which all regression coefficients are assumed to have a common variance. Thus, for an additive model, all markers with the same allele frequency explain the same proportion of the additive variances and have the same shrinkage effect, which makes GBLUP and BRR similar [43].

Bayes A assumes that the markers with the same minor allele frequency contribute differently to genetic variance because the variances in the effect of the markers are heterogeneous; Bayes B is a complement of Bayes A. Hence, Bayes A hypothesizes that that all SNPs have genetic effects and the variance of marker effects should follow the scaled t-distribution, while Bayes B assumes that only a small proportion of SNPs have effects. Bayes B does this by introducing π, the proportion of SNPs with no effect and the variance of marker effects should obey the scaled-t mixture distribution [41]. The models were run using a Gibbs sampler algorithm for a total of 10,000 cycles, discarding the first 5,000 samples for burn-in.

### Design of deep learning models for genomic prediction

In this study, we used the R interface for Keras with the TensorFlow backend for DL models [44]. For the DL-based genomic prediction, we used the CNN and LCNN models. The basic DL structures, number of layers, activation function, optimizer, and number of epochs were designed based on a previous study [33]. The dropout after each layer was used. The rectified linear unit activation function (ReLU) was used with an adaptive estimate of the lower-order moments (Adam) optimizer to minimize the MSE as a loss function [45].

### Bayesian optimization of hyper-parameters

We optimized the hyper-parameters with Bayesian optimization (BO), fitting the Gaussian process using the R package rBayesianOptimization [46]. The optimization bounds were set after some initial evaluations against validation data according to previous studies [19,20,33]. The different hyperparameters that were optimized include kernel size (10 to 50) with one filter, number of units (20 to 200), dropout rate (0 to 0.05), and learning rate (0.00001 to 0.001), and batch size (16 to 512). In CNN and LCNN, the BO used 80% of the training data, where 80% of this dataset was used for optimizing the hyper-parameters and the remaining 20% for validation using Keras independent split validation functions. The number of epochs was set to 50 for each training session.

We set 70 iterations with 20 random initial points and 30 iterations for optimization using the upper confidence bound as an acquisition function in BO. The stochastic sampling of mini-batches in stochastic gradient descent-based algorithms introduces uncertainty, which leads to parameter fluctuations between iterations [21]. Ensemble methods with the replicates of the same algorithm can achieve better predictive performance than with any of the algorithms alone [31]. In this study, 20 replicates of the BO process were run and the hyperparameters were extracted from the model with the highest test Pearson’s correlation, which was then averaged to obtain model-averaged estimates.

### Predictions using cross-validation

The performance of all models was evaluated using five-fold cross-validation. During validation, 80% of the data were used for model training, and the remaining 20% were used for model testing. In the two DL models, 10 replicates were run for each dataset. Obtaining model-averaged estimates can be achieved by averaging the ensemble predictions. The predictive ability of the different methods was assessed as the correlation between predicted genomic breeding values and observed phenotypic values together with the MSE.

## RESULTS AND DISCUSSION

### Estimation of variance components and genetic parameters

The variance components of the two traits are shown in Table 1. For LNL and LPP, the additive genetic component value was lower than those of the dominance and epistatic genetic components. In particular, the epistasis component was higher than those of the additive and dominance components in both LNL (0.19±0.03) and LPP (30.8±7.1). In contrast to the findings of Vitezica et al [47], who reported that the additive component of variance for litter size (i.e., total number of piglets born per litter) in swine was much larger than the dominance and epistatic components, we found that the non-additive components accounted for a substantial proportion of the total genetic variance components for the two SLP phenotypes.

The narrow sense of heritability estimates was low, ranging from 0.06±0.02 (LNL) to 0.10±0.02 (LPP). However, the broad sense of heritability estimates, which included the dominance and epistasis components, were high, ranging from 0.41±0.04 (LPP) to 0.53±0.05 (LNL). Therefore, the non-additive genetic components accounted for a greater fraction of the phenotypic variance components of LNL and LPP than that of the additive genetic component.

There was a high phenotypic (*r*_p_ = 0.70±0.01) and genetic (*r*_g_ = 0.74±0.04). correlations between the two SLP-related traits, suggesting that LNL measurements contain information on LPP vice versa. Bivariate genomic selection approaches take advantage of this information derived from genetic correlations to enhance genomic prediction accuracy [48].

### Hyper-parameters tuning

We optimized hyper-parameters with Gaussian process-based BO, an unbiased and systemic approach which avoids the failure to appropriately scan a large parameter space [31,49], and obtained optimal parameters using ensemble methods by averaging the replicated algorithm. All the hyperparameters for the DL methods chosen in this study are listed in Table 2. In the convolution layer, the selected kernel sizes were 28 and 25 for CNN and LCNN, respectively. The number of fully connected layers was 86 and 91 for the CNN and LCNN, respectively. The dropout rate was 0.28 for the CNN, and 0.34 for the LCNN. The learning rate was 0.0004 for CNN and LCNN.

### Cross-validation for traits

**Figure 1:**
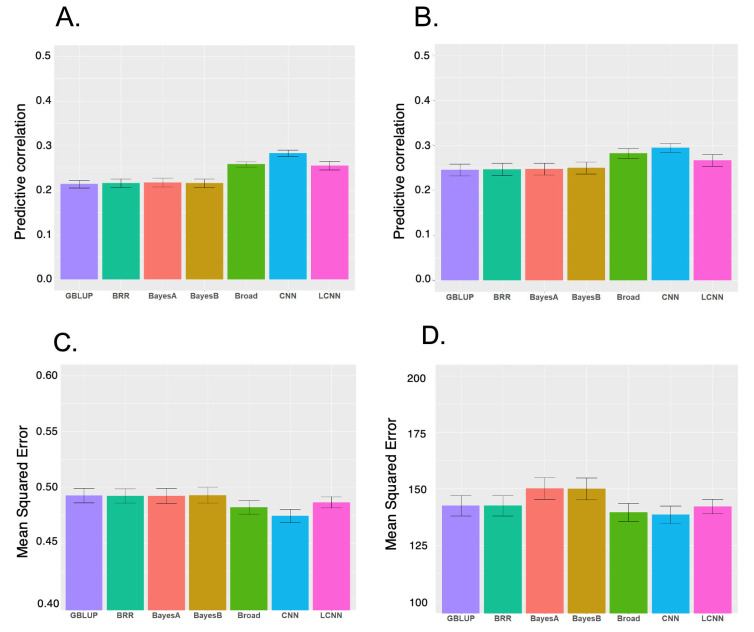
Means and standard errors of predictive performance (predictive correlation and mean squared error) for different genomic prediction models obtained from 5-fold cross-validations. (A) Predictive correlation for lifetime number of litter (LNL). (B) Predictive correlation for lifetime pig production (LPP). (C) Mean squared error for LNL. (D) Mean squared error for LPP. GBLUP, genomic best linear unbiased prediction using additive effect only; BRR, Bayesian ridge regression using additive effect only; Broad: GBLUP using additive, dominance and epistatic effects; CNN, convolutional neural networks; LCNN, local convolutional neural networks.

We evaluated six models for predicting LNL and LPP using five-fold cross-validation (Figure 1). Among the models for the two traits, CNN had the highest performance, followed by LCNN. For LNL, CNN had an outstanding performance for LPP, with the highest predictive correlation (CNN 0.28, LCNN 0.26 and linear models 0.21 to 0.22). The CNN with the lowest MSE (0.47) outperformed the LCNN (0.49) and linear models (approximately 0.49). For LPP, the highest predictive correlation was produced by CNN (0.29), followed by LCNN (0.27), and linear models (0.25). Under MSE, CNN (139), and LCNN (142) outperformed other linear models (BRR/GBLUP 143 and Bayes B/Bayes A 150) (Figure 1). Therefore, CNN and LCNN improved the prediction accuracy and error compared with the results obtained using the linear model-based genomic prediction methods. Additionally, we performed GBLUP with additive, dominance and epistasis effects. The accuracy of this model increased compared to the model with only additive effects, but it was still inferior to CNN. Similarly, the MSE of the broad model decreased compared to the additive model, but it was still higher than CNN (Figure 1).

The prediction performance obtained with GBLUP was similar to those of other Bayesian linear models and slightly better in some cases, indicating that LNL and LPP are most likely controlled by their polygenic nature rather than the large quantitative trait loci effects of particular chromosome regions, and CNN is a robust method for the prediction of these traits. Previously, LCNN was recommended for genomic prediction as a region-specific filter is more consistent with the prior genetic knowledge on the genetic architecture of traits than CNN [33]. In this study, the LCNN had a higher performance than that of linear models in both predictive correlations and MSE for the two traits in this study. However, the performance of LCNN is lower than that of CNN, indicating that applying different filters by marker regions implemented in the LCNN did not improve the predictive performance of the two SLP-related traits.

Two factors could help the predictive performance of convolution-based models (i.e., CNN and LCNN) to outperform linear models in this study. The first factor is the robustness of CNN and LCNN models on the nonlinear patterns in the genetic architecture of traits of interest [18,19]. For example, if a trait has the genetic architecture described by both linear (additivity) and nonlinear patterns (dominance, epistasis), convolution-based models can be robust in the genomic prediction, as shown in Figure 1. In this study, LNL and LPP exhibited a higher broad sense of heritability than the narrow sense of heritability, indicating a substantial contribution of the nonlinear genetic effects on the phenotypic variation of the two traits in pigs. In addition, the CNN and LCNN models can accommodate the correlation between nearby SNP markers (i.e., linkage disequilibrium) via a mathematical operation named convolution [19].

The second factor is the application of a bivariate model for the genomic prediction. The multi-trait models for conventional genomic selection improve prediction accuracy, and genetic correlation between traits is the basis for the advantage of multi-trait models [47]. The LNL and LPP were very similar in genetic architecture, with a strong genetic correlation between them [7]. The genetic correlation coefficient obtained in this study was high. For the DL model, Sandhu et al [25] reported that multi-trait models increased prediction accuracy in genomic selection. Another study using simulation data also reported that in convolution models, the higher the genetic correlation between traits, the higher the prediction accuracy for each trait [33]. Deep learning has great promise for livestock breeding, but its computational burden is a major challenge. Although Table 3 does not show a significant computational burden for DL-based genomic prediction for the study dataset, advances in software and hardware will be required to address this challenge in the future, especially as the number of animals exceeds the number of SNPs. One obvious challenge is that adding data to a DL model typically requires retraining the model, which can be computationally expensive, especially for large datasets. In contrast, multiple regression-based approaches such as BRR, Bayes A, and Bayes B can use marker-level solutions without re-estimation by adding trained data. Another challenge is that the results of BO for DL models may vary depending on the training dataset. This is because DL models are more complex than multiple regression-based models and can be more sensitive to the specific data used for training. Despite these challenges, DL has the potential to become a more practical tool for livestock breeding as advances in software and hardware continue.

## CONCLUSION

In this study, we showed that SLP-related traits are mainly influenced by the polygenic nature and nonlinear interactions of genetic components. CNN is a robust method for predicting genomic breeding values of the two traits. Furthermore, we provide optimized hyperparameters using the Bayesian ensemble approach for the DL models. Our results also suggest that the convolution models with the bivariate method could account for the genetic correlation between bivariate and nonlinear genetic interactions (dominance and epistasis) using filters and neurons. Hence, DL approach can outperform linear model-based genomic prediction approaches for traits with strong non-additive interactions.

## Figures and Tables

**Table 1:** Mean and standard error of variance components and heritabilities

Variance component	LNL	LPP
V_a_	0.03±0.01	14.3±3.2
V_d_	0.06±0.01	17.9±3.9
V_i_	0.19±0.03	30.8±7.1
V_a_+V_d_+V_i_	0.28±0.03	63.6±7.4
V_R_	0.25±0.03	90.1±6.3
V_P_	0.53±0.01	153.7±3.9
h^2^ (narrow sense)	0.06±0.02	0.10±0.02
h^2^ (broad sense)	0.53±0.05	0.41±0.04

LNL, lifetime number of litter; LPP, lifetime pig production; V_a_, additive variance; V_d_, dominance variance; V_i_, additive×additive epistasis variance; V_R_, residual variance; V_P_, phenotypic variance.

**Table 2:** Hyperparameters for deep learning models

Hyperparameters (bounds)	CNN	LCNN
Convolution layer		
Number of layers (fixed)	1	1
Number of filters (fixed)	1	1
Kernel size and strides (10 to 50)	28	25
Fully-connected layer		
Number of layers (fixed)	2	2
Number of neurons (20 to 200)	86	91
Activation (fixed)	ReLU	ReLU
Dropout rate (0 to 0.5)	0.28	0.34
Optimizer (fixed)	Adam	Adam
Learning rate (0.00001 to 0.001)	0.0004	0.0004
Batch size (16 to 512 for only MLP)	32	32
Epochs (fixed)	50	50

CNN, convolutional neural networks; LCNN, local convolutional neural networks; ReLU, rectified linear unit activation function.

**Table 3:** Computational time comparison of genomic prediction models^1)^

Item	Genomic prediction models						

	GBLUP	BRR	BayesA	BayesB	Broad	CNN	LCNN
Runtime (min)	65	160	357	350	115	34	39

GBLUP, genomic best linear unbiased prediction; BRR, Bayesian ridge regression; CNN, convolutional neural networks; LCNN, local convolutional neural networks.

1) CPU, Intel Core i5-8500 CPU@3.00 GHz; GPU, NVIDIA RTX 3080.
